# Different impacts of adipose tissue dynamics on prognosis in patients with resectable locally advanced rectal cancer treated with and without neoadjuvant treatment

**DOI:** 10.3389/fonc.2024.1421651

**Published:** 2024-08-01

**Authors:** Weiyan Huang, Zhichao Feng, Mengtian Ma, Fulong Song, Shumin Zeng, Fang Shao, Xiaoping Yu, Pengfei Rong, Jianqiang Chen

**Affiliations:** ^1^ Department of Radiology, The First Affiliated Hospital of Hainan Medical University, Haikou, China; ^2^ Department of Radiology, The Third Xiangya Hospital, Central South University, Changsha, China; ^3^ Department of Diagnostic Radiology, Hunan Cancer Hospital, Changsha, China

**Keywords:** locally advanced rectal cancer, neoadjuvant chemoradiotherapy, adipose tissue, prognosis, computed tomography

## Abstract

**Background:**

Body composition is recognized to be associated with clinical outcomes in patients with locally advanced rectal cancer (LARC). This study aimed to determine the prognostic role of regional adipose tissue distribution in patients with resectable LARC treated with or without neoadjuvant chemoradiotherapy (nCRT).

**Methods:**

This retrospective study included 281 consecutive patients who underwent radical surgery for LARC with or without preoperative nCRT between 2013 and 2019. Patients underwent contrast-enhanced CT scans before nCRT and before surgery. Visceral adipose tissue (VAT), abdominal subcutaneous adipose tissue (aSAT), and gluteal subcutaneous adipose tissue (gSAT) were quantified on the CT images. The association of adipose tissue distribution with progression-free survival (PFS) was analyzed using Cox proportional hazards analysis.

**Results:**

A total of 102 nCRT-treated and 179 primarily resected patients were included. During a median follow-up period of 24 months, 74 (26.3%) patients experienced local recurrence or metastasis. Multivariable analysis showed that VAT was associated with PFS in all patients (hazard ratio [HR] 1.28, 95% confidence interval [CI] 1.04–1.57; *P* = 0.021). This association was only maintained in primarily resected patients (HR 1.31, 95% CI 1.02–1.69; *P* = 0.037). For patients receiving preoperative nCRT, VAT was not significantly associated with PFS, while the dynamic change in gSAT (ΔgSAT) between nCRT and surgery was associated with PFS (HR 0.43, 95%CI 0.27–0.69, *P* = 0.001).

**Conclusion:**

Visceral obesity is an adverse prognostic factor in patients with resectable LARC treated by primary resection, while increased gluteal subcutaneous adiposity during preoperative nCRT may indicate favorable clinical outcomes.

## 1 Introduction

The global incidence of locally advanced rectal cancer (LARC) is on the rise, with a high risk of postoperative recurrence or distant metastasis (1, 2). Surgical excision has been the basis of LARC treatment. In recent years, with the development of multidisciplinary comprehensive therapy concepts and medical technology, preoperative neoadjuvant chemoradiotherapy (nCRT) has been widely used for resectable LARC (3). However, regardless of the treatment methods, 25% to 30% of patients with LARC experience a distant relapse after radical surgery in clinical practice (4). Therefore, identifying the risk predictors of recurrence or distant metastasis for resectable LARC may help to screen high-risk individuals to improve the prognosis by providing active surveillance or early intervention.

Obesity, sarcopenia, and abnormal distribution of adipose tissue have been found to be negative prognostic factors for patients with LARC (5–7). Excess abdominal adipose tissue can cause serial obesity-related metabolic disorders, including insulin resistance, adipokine perturbation, and chronic inflammation, which promote carcinogenesis and cancer progression (8, 9). Moreover, the difference in the intracellular development of the adipocyte population also results in opposite effects of upper and lower-body obesity on the immune and metabolic capacities (10). Previous studies tended to investigate the impact of visceral adipose tissue on the clinical outcomes in patients with resectable LARC at a single time point, revealing that visceral obesity was associated with shorter overall survival, increased risk of postoperative complications and increased length of stay in patients undergoing surgery in LARC treated with nCRT (11–13). However, studies evaluating the association between dynamic changes in regional adipose tissue and prognosis in patients with resectable LARC are still lacking. We hypothesized that the impacts of adipose tissue distribution on prognosis between patients treated with primary resection and those receiving preoperative nCRT differed, and the dynamic changes in regional adipose tissue during nCRT were associated with prognosis.

Therefore, this study evaluated the prognostic role of regional adipose tissue in patients with resectable LARC treated with or without nCRT. Meanwhile, we further investigated the potential impact of nCRT on adipose tissue redistribution.

## 2 Methods

### 2.1 Study population

The study was approved by the institutional review board of The Third Xiangya Hospital, Central South University (Changsha, China) and Hunan Cancer Hospital (Changsha, China), and the requirement to obtain informed consent from patients was waived. This retrospective study included 281 consecutive patients who underwent radical surgery for LARC in The Third Xiangya Hospital, Central South University and Hunan Cancer Hospital from July 2013 to July 2019. LARC is defined as T3/T4 primary tumors or node-positive malignancies with no distant metastases (14). The diagnosis of LARC was based on the pathological examination of the tissue taken from the rectum. Patients with LARC included in the study were divided into patients treated with primary resection and those receiving preoperative nCRT by two different treatment methods. Patients were included if they satisfied the following criteria: (a) all patients underwent radical surgery and were confirmed pathologically; (b) patients underwent contrast-enhanced CT scans before nCRT and before surgery; (c) clinical data and pathology results were available. The exclusion criteria were as follows: (a) patients had a history of preoperative treatment other than nCRT; (b) CT image quality was poor; (c) nCRT treatment was incomplete.

Baseline demographic information, laboratory tests, and pathological results were obtained from electronic medical records, which included age, gender, height, weight, body mass index (BMI, weight divided by height squared), neutrophil, lymphocyte, monocyte, albumin, carcinoembryonic antigen (CEA), and TNM tumor stage.

### 2.2 Adipose tissue quantification

Baseline enhanced CT venous phase images of patients at the level of the third vertebra (L3) and ischial tuberosity were obtained for adipose tissue measurement from the PACS imaging system (15, 16). Each selected CT image was assessed by a single reviewer who was blinded to the clinical, pathological, and outcome data, using opensource software (NIH ImageJ version 1.51j8, https://imagej.nih.gov/ij/), which has previously been validated to provide reliable measurements (17). Standard radiodensity thresholds measured in Hounsfield units (HU) were used to quantify the visceral adipose tissue area (VAT), abdominal subcutaneous adipose tissue area (aSAT), and gluteal subcutaneous adipose tissue area (gSAT). Thresholds for VAT are between –150 and –50 HU, and thresholds for aSAT and gSAT are between –190 and –30 HU (15). Visceral obesity was defined as the VAT area greater than 100cm^2^ (18). Patients treated with preoperative nCRT underwent enhanced CT scans before nCRT and preoperative, and patients undergoing primarily resection underwent preoperative enhanced CT scan. The longitudinal change of adipose tissue in nCRT patients was expressed by the rate of change, which was the change of adipose tissue area (Δ) (the difference between preoperative and pre-nCRT) divided by the time interval (day) (Equation 1).


(1) $$\begin{array}{c} \text{ΔVAT};\text{aSAT};\text{gSAT}\\ \;=((\text{preoperative CT area}–\text{ pre}\\ \;-\text{nCRT CT area})\;(〖cm〗\hat{\;}2))\\ \;/((\text{time between preoperative and pre}-\text{nCRT CT})\;(\text{day})) \end{array}$$

### 2.3 Treatment procedures

Primarily resected patients were treated by radical surgery using laparoscopic or open routes. Radical surgery included low anterior resection, abdominoperineal resection, and extended Hartmann procedure. nCRT treated patients underwent radical surgery at 5–12 weeks after completing continuous nCRT. nCRT regimens were as follows: patients were treated with long-term radiotherapy/capecitabine or long-term radiotherapy/continuous 5-Fu or long-term radiotherapy/5-Fu/LV. The recommended irradiation dose was 45–50Gy, divided into 25–28 times, and multi-field irradiation (usually 3–4 field technique) was adopted (19).

### 2.4 Follow-up and outcomes

Patients were followed up every 3–6 months after surgery for surveillance imaging (computed tomography chest imaging as well as abdominal and pelvic imaging on computed tomography, magnetic resonance imaging, or positron emission tomography) until disease progression, the end of the study period, or loss to follow-up. Analyses in this current report are based on updated clinical data and patient follow-up as of July 30, 2022. The primary outcome in this study was progression-free survival (PFS), which was defined as time from surgery to first occurrence of documented disease progression. Patients without an event were censored at their last disease evaluation date.

### 2.5 Statistical analysis

Statistical analysis was performed using the statistical software R (the R Foundation for Statistical Computing; Version 4.1.1; https://www.r-project.org/). Continuous variables were expressed as means and standard deviations (SDs), and categorical variables were expressed as numbers and percentages. Continuous variables were compared using the independent two samples t-test, and categorical variables were compared using the chi-square test or Fisher exact test. Survival curves were constructed by the Kaplan-Meier method, and the log-rank test was performed to compare the difference between groups. The Cox proportional hazard model was adopted for univariable and multivariable analyses of potential risk factors associated with PFS, and hazard ratios (HRs) and 95% confidence intervals (CIs) were calculated. The time-dependent receiver operating characteristic (ROC) curve was used to evaluate the predictive ability of variables for survival outcomes. A *P* < 0.05 was considered statistically significant and all reported *P* values were two-sided.

## 3 Results

### 3.1 Patient characteristics

From July 2013 to July 2019, a total of 281 patients with LARC (median age, 54.65 years; 190 males) were included, consisting of 102 patients who received preoperative nCRT (mean age, 51.87 years; 67 men) and 179 patients who underwent primary resection (mean age 56.23 years; 123 men) (**Supplementary Figure 1**). The demographic and clinical characteristics are shown in **Table 1**. The two groups showed significant differences in age (*P* = 0.001), monocyte count (*P* = 0.001), albumin level (*P* = 0.008), CEA level (*P* = 0.023), clinical stage (*P* < 0.001), lymph node stage (*P* < 0.001) and postoperative TNM stage (*P* < 0.001). No differences were found in other characteristics.

**Table 1:** Baseline clinical characteristics of patients.

Characteristics	Total (*n* = 281)	nCRT (*n* = 102)	Primary Resection (*n* = 179)	*P*
Age, yrs, mean (SD)	54.65 (10.38)	51.87 (8.47)	56.23 (11.03)	0.001**
Gender	281	102	179	0.697
Male	190 (67.6%)	67 (65.7%)	123 (68.7%)	
Female	91 (32.4%)	35 (34.3%)	56 (31.3%)	
BMI, kg/m^2^, mean (SD)	22.55 (2.74)	22.58 (3.10)	22.54 (2.53)	0.914
NLR, mean (SD), mean (SD)	2.72 (2.28)	2.70 (2.31)	2.72 (2.27)	0.937
Monocytes,10^9^/L	0.45 (0.17)	0.49 (0.18)	0.42 (0.16)	0.001**
Albumin, g/L, mean (SD)	41.63 (4.50)	42.57 (3.90)	41.09 (4.74)	0.008**
CEA ng/ml, mean (SD)	10.45 (14.19)	7.91 (11.28)	11.90 (15.45)	0.023 *
Clinical stage				<0.001***
II	92 (32.7%)	15 (14.7%)	77 (43.0%)	
III	189 (67.3%)	87 (85.3%)	102 (57.0%)	
Clinical T stage				0.515
T2	13 (4.6%)	4 (3.9%)	9 (5.0%)	
T3	159 (56.6%)	54 (52.9%)	105 (58.7%)	
T4	109 (38.8%)	44 (43.1%)	65 (36.3%)	
Clinical N stage				<0.001***
N0	92 (32.7%)	15 (14.7%)	77 (43.0%)	
N1	76 (27.1%)	21 (20.6%)	55 (30.7%)	
N2	113 (40.2%)	66 (64.7%)	47 (26.3%)	
ypT/pT stage				<0.001***
T0	15 (5.3%)	15 (14.7%)	–	
T1	4 (1.5%)	4 (3.9%)	–	
T2	51 (18.1%)	42 (41.2%)	9 (5.0%)	
T3	140 (49.8%)	35 (34.3%)	105 (58.7%)	
T4	71 (25.3%)	6 (5.9%)	65 (36.3%)	
ypN/pN stage				<0.001***
N0	150 (53.4%)	73 (71.6%)	77 (43.0%)	
N1	74 (26.3%)	19 (18.6%)	55 (30.7%)	
N2	57 (20.3%)	10 (9.8%)	47 (26.3%)	
VAT, cm^2^	83.02 (56.78)	89.19 (67.22)	79.51 (49.72)	0.170
aSAT, cm^2^	98.41 (51.98)	106.07 (58.28)	94.04 (47.64)	0.062
gSAT, cm^2^	130.64 (49.90)	127.85 (56.80)	132.23 (45.60)	0.480
VSR	0.93 (0.63)	0.90 (0.62)	0.95 (0.64)	0.569

Continuous variables were expressed as mean and standard deviation (SD), categorical variables were expressed as numbers (percentages).

BMI, body mass index; NLR, Neutrophil to Lymphocyte Ratio; SD, standard deviation; VAT, visceral adipose tissue; aSAT, abdominal subcutaneous adipose tissue; gSAT, gluteal subcutaneous adipose tissue; VSR, the ratio of VAT to aSAT. “*” represents a p-value of less than 0.05, “**” represents a p-value of less than 0.01, “***” represents a p-value of less than 0.0001.

### 3.2 Association between visceral obesity and survival

**Figure 1:**
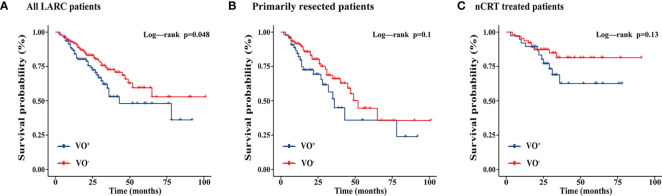
Kaplan-Meier curves showing the effects of visceral adipose tissue on progression-free survival of all LARC patients, primarily resected patients, and nCRT treated patients. nCRT, neoadjuvant chemoradiotherapy; LARC, locally advanced rectal cancer; VO, Visceral obesity **(A)** All LARC patients; **(B)** Primarily resected patients; **(C)** nCRT treated patients.

During a median follow-up of 24 months (IQR, 13.0–34.5 months), 74 (26.3%) patients experienced local recurrence or distant metastases, including 21 patients treated with preoperative nCRT and 53 patients treated with primary resection. Kaplan-Meier curves showed that visceral obesity was associated with an increased risk of local recurrence or metastasis in all patients (*P* = 0.048). However, this association was not maintained in patients treated with primary resection (*P* = 0.1) and in patients treated with preoperative nCRT (*P* = 0.13) (**Figure 1**). **Supplementary Figure 2** shows the CT results of adipose tissue measured at L3 and TI levels in patients with LARC.

### 3.3 Association between regional adipose tissue and survival

To further explore the prognostic impact of regional adipose tissue on PFS, we performed a cox regression analysis. Univariable analysis revealed that VAT (HR 1.34, 95% CI 1.11–1.62; *P* = 0.002) and aSAT (HR 1.27, 95% CI 1.05–1.54, *P* = 0.016) were associated with poor prognosis in all patients. Multivariable analysis confirmed that VAT (HR 1.28, 95% CI 1.04–1.57, *P* = 0.021) was an important prognostic factor (**Table 2**).

**Table 2:** Univariable and multivariable Cox regression analysis of predictors associated with PFS in all patients.

Variables	Univariable HR (95% CI)	*P*	Multivariable HR (95% CI)	*P*
Age, yrs	1.00 (0.98-1.03)	0.777		
Male gender	0.86 (0.53-1.40)	0.553		
BMI, kg/m^2^	1.08 (1.00-1.17)	0.050		
NLR	1.03 (0.94-1.13)	0.477		
Monocytes,10^9^/L	0.29 (0.07-1.25)	0.098		
Albumin, g/L	1.05 (0.99-1.10)	0.110		
CEA ng/ml	1.00 (0.99-1.02)	0.815		
Clinical stage				
II	Ref	Ref		
III	1.18 (0.69-2.02)	0.541		
T stage				
T2	Ref	Ref		
T3	1.31 (0.40-4.30)	0.657		
T4	2.49 (0.76-8.18)	0.133		
N stage				
N0	Ref	Ref		
N1	1.03 (0.54-1.95)	0.928		
N2	1.28 (0.73-2.27)	0.388		
Baseline VAT, cm^2^	1.34 (1.11-1.62)	0.002**	1.28 (1.04-1.57)	0.021*
Baseline aSAT, cm^2^	1.27 (1.05-1.54)	0.016*	1.17 (0.94-1.46)	0.168
Baseline gSAT, cm^2^	1.22 (0.99-1.49)	0.061		
VSR	1.10 (0.87-1.38)	0.427		

BMI, body mass index; NLR, Neutrophil to Lymphocyte Ratio; SD, standard deviation; VAT, visceral adipose tissue; aSAT, abdominal subcutaneous adipose tissue; gSAT, gluteal subcutaneous adipose tissue; VSR, the ratio of VAT to aSAT. “*”represents a p-value of less than 0.05, “**”represents a p-value of less than 0.01, “***” represents a p-value of less than 0.0001.

Univariable analysis showed that pN staging (HR 2.70, 95% CI 1.39–5.26; *P* = 0.003), VAT (HR 1.36, 95% CI 1.11–1.68; *P* = 0.003) and aSAT (HR 1.50, 95% CI 1.15–1.96, *P* = 0.003) were significantly associated with PFS in patients with primary resection. They remained as predictive factors for poorer prognosis (*P* = 0.005, *P* = 0.037, and *P* = 0.042, respectively) in multivariable analysis (**Supplementary Table 1**).

However, univariable analysis showed that no statistically significant association between VAT (*P* = 0.071) and patient prognosis in patients treated with preoperative nCRT, while the ΔgSAT stage (HR 0.48, 95% CI 0.31–0.75, *P* = 0.001) was an important predictor of PFS. In multivariable analysis, ΔgSAT (HR 0.43, 95% CI 0.27–0.69, *P* = 0.001) was still a positive prognostic factor (**Table 3**).

**Table 3:** Univariable and multivariable Cox regression analysis of predictors associated with PFS in patients receiving preoperative nCRT.

Variables	Univariable HR (95% CI)	*P*	Multivariable HR (95% CI)	*P*
Age, yrs	0.97 (0.92-1.01)	0.176		
Male gender	0.79 (0.33-1.90)	0.597		
BMI, kg/m^2^	1.11 (0.97-1.28)	0.123		
NLR	1.13 (0.99-1.28)	0.078		
Monocytes,10^9^/L	0.12 (0.01-1.87)	0.129		
Albumin, g/L	1.10 (0.99-1.22)	0.086		
CEA ng/ml	1.00 (0.97-1.04)	0.807		
Clinical TNM stage				
II	Ref	Ref		
III	0.57 (0.19-1.72)	0.323		
ypT stage				
T0、Tis	Ref	Ref	Ref	Ref
T1	0 (0-Inf)	0.998	0.00 (0.00-Inf)	0.998
T2	1.49 (0.32-7.02)	0.614	1.28 (0.26-6.26)	0.758
T3	1.89 (0.39-9.13)	0.427	1.68 (0.31-9.02)	0.545
T4	12.38 (2.15-71.16)	0.005**	8.27 (1.18-58.17)	0.034*
ypN stage				
N0	Ref	Ref	Ref	Ref
N1	2.98 (1.15-7.70)	0.024*	1.88 (0.63-5.58)	0.257
N2	2.11 (0.59-7.58)	0.251	1.69 (0.41-6.87)	0.464
baseline VAT, cm^2^	1.41 (0.97-2.04)	0.071		
baseline aSAT, cm^2^	1.26 (0.87-1.83)	0.222		
baseline gSAT, cm^2^	1.23 (0.83-1.80)	0.300		
VSR	1.11 (0.74-1.65)	0.613		
ΔVAT, cm^2^/d	0.89 (0.62-1.27)	0.519		
ΔaSAT, cm^2^/d	0.73 (0.47-1.16)	0.187		
ΔgSAT, cm^2^/d	0.48 (0.31-0.75)	0.001***	0.43 (0.27-0.69)	0.001***


BMI, body mass index; NLR, Neutrophil to Lymphocyte Ratio; SD, standard deviation; VAT, visceral adipose tissue; aSAT, abdominal subcutaneous adipose tissue; gSAT, gluteal subcutaneous adipose tissue; VSR, the ratio of VAT to aSAT; ΔVAT, the change rate of VAT; ΔaSAT, the change rate of aSAT; ΔgSAT, the change rate of gSAT. “*” represents a p-value of less than 0.05, “**” represents a p-value of less than 0.01, “***” represents a p-value of less than 0.0001.

### 3.4 Prognostic impact of gluteal adipose tissue redistribution in nCRT-treated patients

**Figure 2:**
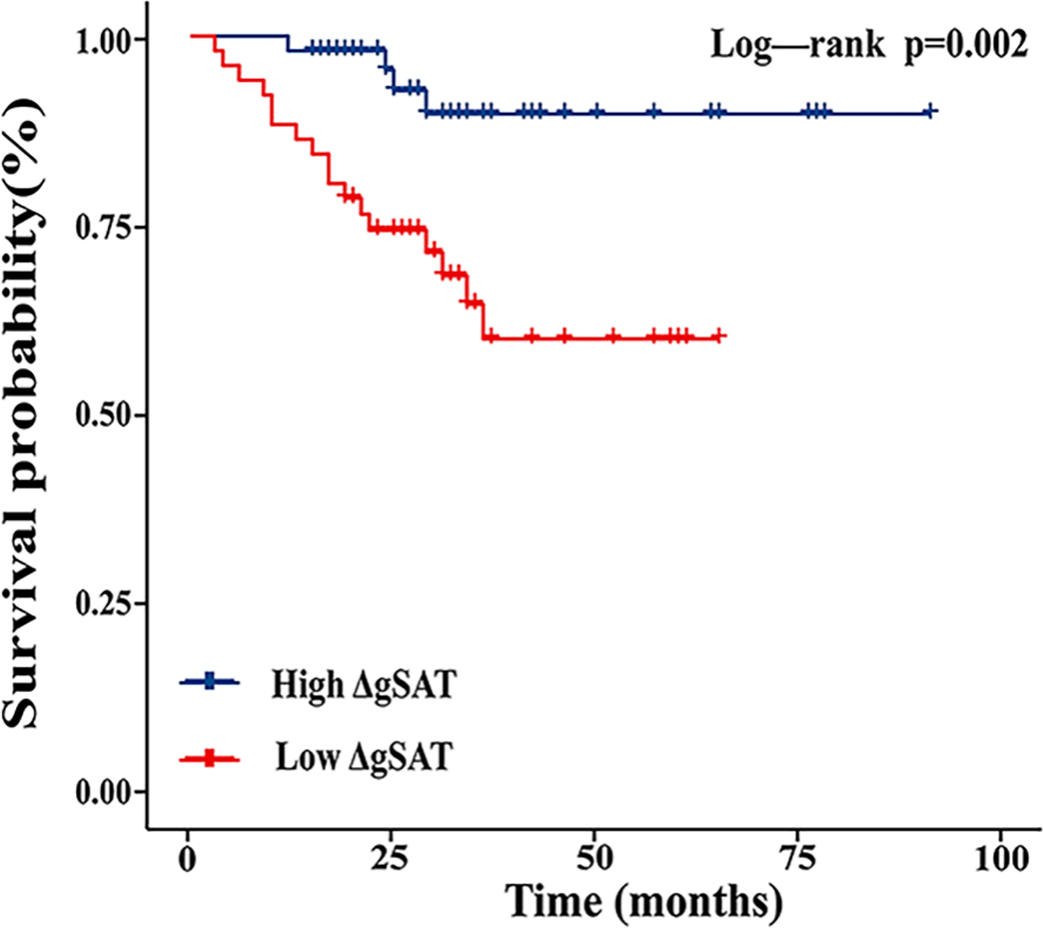
Kaplan-Meier curves showing the effect of gluteal subcutaneous adipose tissue change rate (ΔgSAT) on progression-free survival in patients receiving preoperative nCRT.

To further evaluate the relationship between gluteal adipose tissue and prognosis in nCRT patients, we performed Kaplan-Meier curve analysis and time-dependent ROC curve analysis. Kaplan-Meier survival analysis showed (**Figure 2**) that high ΔgSAT was associated with a reduced risk of local recurrence or metastasis (*P* = 0.002). Patients with ΔgSAT above the corresponding median were classified as high ΔgSAT.

**Figure 3:**
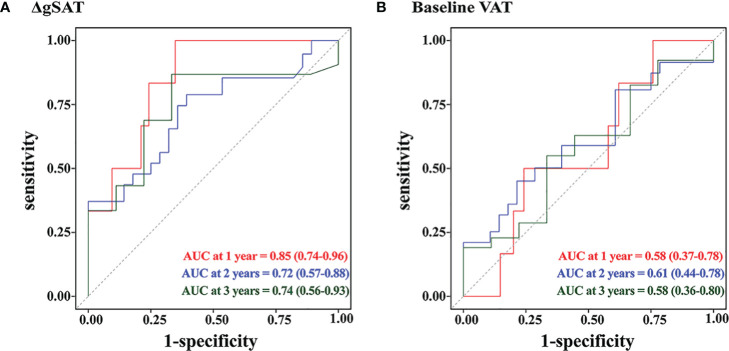
Time-dependent ROC analysis in patients with LARC treated by nCRT. **(A)** ΔgSAT; **(B)** Baseline VAT.

Time-dependent ROC curve analysis in patients treated with preoperative nCRT showed that the area under the 1, 2, and 3-year curves for ΔgSAT were 0.85 (95% CI 0.73–0.96), 0.72 (95% CI: 0.56–0.87) and 0.74 (95% CI: 0.56–0.93) respectively. The area under the 1, 2, and 3-year curves for VAT were 0.58 (95% CI 0.37–0.78), 0.61 (95% CI 0.44–0.78), and 0.58 (95% CI:0.36–0.80), respectively (**Figure 3**). ΔgSAT showed a better ability to predict early local recurrence and distant metastasis, compared with baseline VAT.

## 4 Discussion

This study has evaluated the prognostic effect of regional adipose tissue on patients with LARC. Meanwhile, we have done further investigation adipose tissue changes in different groups and the redistribution effect on adipose tissue from nCRT. In our study, visceral obesity was a negative prognostic predictor in patients with LARC, while in patients treated with preoperative nCRT, ΔgSAT was associated with significantly reduction in cancer recurrence and distant metastasis. It indicated that ΔgSAT was a positive prognostic factor, and nCRT might play a role in the redistribution of body adipose tissue. Based on the research results, the accumulation of preoperative gSAT had a protective effect on the prognosis of LARC patients, which would help establish a preoperative nCRT metabolic risk assessment for LARC and improve the prognosis of the patient.

Some studies have investigated the effects of VAT on patients undergoing the surgery for bowel cancer. Basile (20) et al. reported a significant association between high VAT and poor prognosis of metastatic colorectal cancer. Guiu (21) et al. demonstrated that VAT was an independent predictive biomarker ensued from the first-line bevacizumab-based treatment in metastatic colorectal cancer. We found that VAT was a negative prognostic factor in patients with resectable LARC, confirming the survival rate between VAT and LARC in other studies.

In addition, so far only a few studies have evaluated changes in body composition of cancer patients during nCRT. Yip et al. (22) shown that after nCRT for esophageal cancer, differential loss of visceral to subcutaneous adipose tissue ratio associated with the risk of circumferential resection margin positivity. Liu (23) et al. showed that pre-nCRT low muscle density and loss of total abdominal fat area were related to a high incidence of short- and long-term ileus, respectively. Heus (12) et al. found that visceral obesity related with more complications and a longer length of stay in rectal cancer surgery, but during nCRT VAT area was not affected by chemoradiotherapy. In our study, the change rate of adipose tissue was used to represent the dynamic change of adipose tissue, which could more intuitively see the change trend of adipose tissue. Meanwhile, considering the developmental and functional differences between the upper body adipose depot and the lower body adipose tissue, we have analyzed the relationship between the lower body adipose tissue and LARC. We focused on the effect of nCRT on adipose tissue distribution, and the relevance of dynamic changes in adipose tissue to the prognosis of LARC. We found that the ratio of VAT to aSAT (VSR), ΔVAT, and ΔaSAT were found to have no significant correlation with the prognosis of nCRT treated patients, but ΔgSAT had an obvious predictive value. We have further analyzed ΔgSAT and classified by the median, to find high ΔgSAT have associated with the lower recurrence and distant metastasis of LACR.

Compared with VAT, ΔgSAT was a positive prognostic factor for LARC, due to differences in microvascular and metabolic characteristics resulted from different patterns of adipokine secretion and endocrine function between upper and lower body fat (24, 25). The Intra-abdominal adipose depot was related to the viscera. VAT had strong lipolytic activity and could release more free fatty acids, which could induce insulin resistance, inflammation and oxidative stress through lipid mediators such as ceramides, increasing the risk of cancer (26, 27). Furthermore, VAT could produce higher proinflammatory cytokines and immune cells to induce tumor occurrence and diffusion (28). The reduced lipid turnover of lower body adipose storage could accommodate redistributed adipose tissue and show fewer signs of inflammatory damage (25). What’s more, the gluteal vascular network was not as rich as the abdomen, the blood flow being low, and the action rate of hormone sensitive lipase being also low, causing a lower overall fatty acid release rate and uptake rate than the abdomen, the energy supply reflex reduced, which were opposite to metabolic effects of the abdominal adipose (29, 30).

We hypothesized that nCRT affected the distribution of adipose tissue in the buttocks and abdomen. For patients with adipose tissue metastases from the abdomen to the gluteal, the overall release of fatty acids and pro-inflammatory cytokines was less than in patients without fat transfer, which caused a decrease in tumor oxidative stress and an increase in the sensitization of tumor cells to radiotherapy and chemotherapy, thereby slowing down the progress of tumor. In view of this, we could help provide quantitative imaging markers to assist patients by monitoring changes in adipose tissue distribution and provide datum for active postoperative monitoring and early intervention in high-risk patients, which could be clinically useful.

During the past years, deep learning (DL) has steadily found its way into the field of medicine and pathology, and tend nowadays to have an expanding role in all fields of medicine. Several studies have found that deep learning advances have the potential to improve the accuracy and validity of CRC detection (31, 32). Deep learning algorithms can accurately predict patients who will have a complete pathological response after nCRT for LARC (33); Deep learning-based body composition can be used to model survival in LARC (34). We will also consider referencing these algorithms in ongoing studies.

Our research has several limitations. First, this study was retrospectively conducted, which might introduce potential selection biases. Second, the results were only applicable to tumors at local clinical progression stage, and could not represent all rectal cancer patients, which needed further verifying in patients with advanced diseases. Third, since the study was a retrospective analysis, other metabolic characteristics related to obesity need to be considered in future prospective studies. However, a large amount of so-called hidden data could be extracted from medical images through radiology, which was helpful in improving diagnostic performance.

In conclusion, visceral obesity is an adverse prognostic factor in patients with resectable LARC treated by primary resection, while increased gluteal subcutaneous adiposity during preoperative nCRT may indicate favorable clinical outcomes. Preoperative nCRT may cause the redistribution of gluteal and abdominal adipose tissue in patients with resectable LARC.

## Data availability statement

The raw data supporting the conclusions of this article will be made available by the authors, without undue reservation.

## Ethics statement

The studies involving humans were approved by Third Xiangya Hospital, Central South University (Changsha, China) and Hunan Cancer Hospital (Changsha, China). The studies were conducted in accordance with the local legislation and institutional requirements. Written informed consent for participation was not required from the participants or the participants’ legal guardians/next of kin in accordance with the national legislation and institutional requirements.

## Author contributions

WH: Conceptualization, Data curation, Formal analysis, Investigation, Methodology, Software, Validation, Writing – original draft, Writing – review & editing. JC: Funding acquisition, Methodology, Supervision, Writing – review & editing. PR: Funding acquisition, Resources, Supervision, Writing – review & editing. ZF: Formal analysis, Methodology, Software, Writing – original draft. MM: Methodology, Software, Writing – original draft. FLS: Data curation, Writing – original draft. SZ: Conceptualization, Writing – original draft. FS: Conceptualization, Writing – original draft. XY: Resources, Writing – review & editing.
