# Supplementary material for: Different impacts of adipose tissue dynamics on prognosis in patients with resectable locally advanced rectal cancer treated with and without neoadjuvant treatment
Source: Front Oncol. 2024 Aug 1;14:1421651. doi: 10.3389/fonc.2024.1421651 (PMC11324464; doi:10.3389/fonc.2024.1421651)
Supplement: Supplementary file 1 [file Table_1.docx]

| **Supplementary Table 1** Univariable and multivariable Cox regression analysis of predictors associated with PFS in primarily resected patients. | | | | | |
| --- | --- | --- | --- | --- | --- |
| Variables | Univariable HR (95% CI) | *P* | Multivariable HR (95% CI) | *P* | |
| Age, yrs | 1.00 (0.98-1.03) | 0.818 |  |  | |
| Male gender | 0.84 (0.47-1.50) | 0.559 |  |  | |
| BMI, kg/m^2^ | 1.06 (0.95-1.17) | 0.296 |  |  | |
| NLR | 0.98 (0.87-1.11) | 0.798 |  |  | |
| Monocytes,10^9^/L | 0.69 (0.12-4.08) | 0.682 |  |  | |
| Albumin, g/L | 1.03 (0.97-1.10) | 0.316 |  |  | |
| CEA ng/ml | 1.00 (0.98-1.01) | 0.862 |  |  | |
| Clinical stage |  |  |  |  | |
| II | Ref | Ref |  |  | |
| III | 1.84 (0.99-3.41) | 0.054 |  |  | |
| pT stage |  |  |  |  | |
| T2 | Ref | Ref |  |  | |
| T3 | 1.92 (0.45-8.30) | 0.380 |  |  | |
| T4 | 4.00 (0.92-17.34) | 0.064 |  |  | |
| pN stage |  |  |  |  | |
| N0 | Ref | Ref | Ref | Ref | |
| N1 | 1.22 (0.59-2.54) | 0.591 | 1.44 (0.68-3.04) | 0.337 | |
| N2 | 2.70 (1.39-5.26) | 0.003** | 2.63 (1.34-5.13) | 0.005** | |
| Baseline VAT, cm^2^ | 1.36 (1.11-1.68) | 0.003** | 1.31 (1.02-1.69) | 0.037* | |
| Baseline aSAT, cm^2^ | 1.50 (1.15-1.96) | 0.003** | 1.37 (1.01-1.85) | 0.042* | |
| Baseline gSAT, cm^2^ | 1.06 (0.79-1.43) | 0.063 |  |  | |
| VSR | 1.29 (0.99-1.69) | 0.682 |  |  | |
| Abbreviations: BMI, body mass index; NLR, Neutrophil to Lymphocyte Ratio; SD, standard deviation; VAT, visceral adipose tissue; aSAT, abdominal subcutaneous adipose tissue; gSAT, gluteal subcutaneous adipose tissue; VSR, the ratio of VAT to aSAT. | | | | |  |
