# Supplementary material for: Different impacts of adipose tissue dynamics on prognosis in patients with resectable locally advanced rectal cancer treated with and without neoadjuvant treatment
Source: Front Oncol. 2024 Aug 1;14:1421651. doi: 10.3389/fonc.2024.1421651 (PMC11324464; doi:10.3389/fonc.2024.1421651)

Supplementary Figure.1 Flowchart of patient selection. nCRT= neoadjuvant chemoradiotherapy; LARC= locally advanced rectal cancer

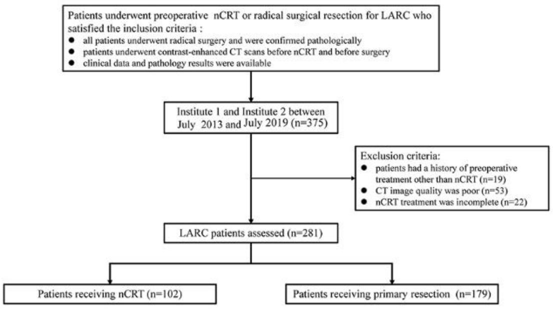

Supplement: Supplementary file 2 [file Image_1.pdf]
