# Supplementary material for: Different impacts of adipose tissue dynamics on prognosis in patients with resectable locally advanced rectal cancer treated with and without neoadjuvant treatment
Source: Front Oncol. 2024 Aug 1;14:1421651. doi: 10.3389/fonc.2024.1421651 (PMC11324464; doi:10.3389/fonc.2024.1421651)

Supplementary Figure 2 Area-based quantification of adipose tissue at the L3 (A, B) and ischial tuberosity (C, D) levels of a 48-year-old female patient.

A. CT images of the venous phase at the L3 level

B. Red: aSAT, abdominal subcutaneous adipose tissue; Green, VAT, visceral adipose tissue

C. CT images of the venous phase at the level of ischial tuberosity

D: Yellow: gSAT, gluteal subcutaneous adipose tissue

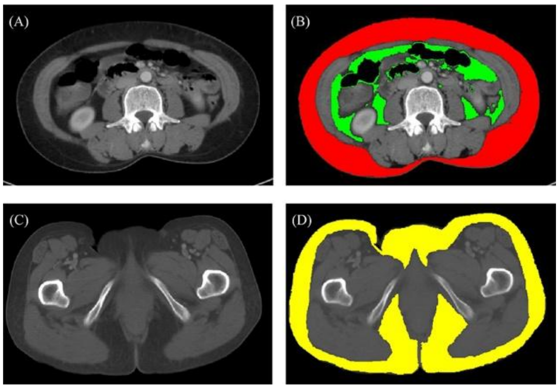

Supplement: Supplementary file 3 [file Image_2.pdf]
